# *Neisseria meningitidis* ST11 Complex Isolates Associated with Nongonococcal Urethritis, Indiana, USA, 2015–2016

**DOI:** 10.3201/eid2302.161434

**Published:** 2017-02

**Authors:** Evelyn Toh, Dharanesh Gangaiah, Byron E. Batteiger, James A. Williams, Janet N. Arno, Albert Tai, Teresa A. Batteiger, David E. Nelson

**Affiliations:** Indiana University School of Medicine, Indianapolis, Indiana, USA (E. Toh, D. Gangaiah, B.E. Batteiger, J.A. Williams, J.N. Arno, T.A. Batteiger, D.E. Nelson);; Tufts University School of Medicine, Boston, Massachusetts, USA (A. Tai).

**Keywords:** Neisseria, urethritis, meningitis, *Neisseria gonorrhoeae*, *Neisseria meningitidis*, sexually transmitted disease, sexually transmitted infections, urethra, genome, bacteria

## Abstract

At a clinic in Indianapolis, Indiana, USA, we observed an increase in *Neisseria gonorrhoeae*–negative men with suspected gonococcal urethritis who had urethral cultures positive for *N. meningitidis*. We describe genomes of 2 of these *N. meningitidis* sequence type 11 complex urethritis isolates. Clinical evidence suggests these isolates may represent an emerging urethrotropic clade.

*Neisseria meningitidis* and *N. gonorrhoeae* are exclusive human pathogens. *N. meningitidis* is a leading cause of sepsis and meningitis, whereas *N. gonorrhoeae* (gonococcus) traditionally causes gonorrhea, a sexually transmitted infection involving the genitals, rectum, and throat. These species usually occupy distinct niches but may cause reciprocal diseases when *N. meningitidis* colonizes the anogenital tract or gonococcus colonizes oropharyngeal mucous membranes (*1*,*2*).

Sporadic cases of meningococcal urethritis have been reported since the 1930s (*3*). It was recognized by the 1970s that urethral *N. meningitidis* infections could be spread by oral sex and were more common in men who have sex with men (*4*). Most *N. meningitidis* urethritis cases identified before 1993 were caused by strains from serogroups A and B (*5*), but outbreaks involving other serogroups and nontypeable strains have been reported (*6*–*10*). These cases have typically presented with purulent urethritis or proctitis with gram-negative intracellular diplococci (GNID) identified by Gram stain of urethral exudates. The current prevalence of meningococcal urethritis is unknown in the United States but was thought to be low (4). However, a recent spike in heterosexual *N. meningitidis* urethritis cases beginning in 2013 in Ohio and Michigan, reported by Bazan et al. in association with the Gonococcal Isolate Surveillance Project, was linked to a nongroupable clonal *N. meningitidis* strain (sequence type 11 [ST11], clonal complex [CC] ET-37) (*11*). Whether *N. meningitidis* ST11 strains are common causes of nongonococcal urethritis or if these strains have adaptations that enhance their virulence for the urethra is unknown because genomes of urethral *N. meningitidis* isolates have not been previously reported.

Beginning in early 2015, we observed that 4 (6%) of 59 men we enrolled in gonococcus treatment trials in our clinic in Indianapolis, Indiana, USA, and who had urethral specimens that were positive on Gram stain tested negative for gonococcus by specific nucleic acid amplification tests (NAATs). Sugar fermentation reaction profiles of the isolates from all 4 of these men suggested that they were infected with *N. meningitidis*. We describe the recent epidemiology of these suspected *N. meningitidis* urethritis cases and the genomes of 2 of these *N. meningitidis* isolates.

## The Study

We enrolled 59 men (age range 20–61 years, median age 31 years) in 3 gonoccocal treatment trials from April 2014 through February 2016 at the Bell Flower Clinic, a public sexually transmitted infections clinic, in Indianapolis, Marion County, Indiana. All of these men had purulent urethral discharge with >10 leukocytes and GNID on Gram stains of urethral exudate. However, 4 men tested negative for gonococcus with specific NAATs (APTIMA Combo 2, Gen-Probe Inc., San Diego, CA, USA; or COBAS 4800, Roche Diagnostics, Indianapolis, IN, USA). All 4 men reported recent vaginal and oral sexual exposures. Urethral cultures yielded growth with colony morphology, oxidase, and Gram stain results consistent with gonococcus, but the isolates fermented glucose and maltose but not lactose, consistent with *N. meningitidis*. A pharyngeal swab from 1 of these men also grew *N. meningitidis*, whereas rectal swab specimens from all 4 were culture negative. All 4 cases responded to investigational gonococcal antibiotic regimens and were culture plus NAAT negative by test of cure assessed at day 7 posttreatment. Two of these isolates (NM1 and NM2), which were susceptible to ampicillin, ceftriaxone, chloramphenicol, levofloxacin and meropenem, were subcultured and confirmed to be *N. meningitidis* by matrix-assisted laser desorption/ionization time-of-flight mass spectrometry.

Whole-genome sequencing of NM1 and NM2 (Technical Appendix) revealed that these 2 isolates closely resembled strains of ST11 and CC11, with fine type PorA VR 1.5–1, PorA2 10–8; FetA3–6 ST11 (CC11). Phylogenetic analysis indicated that NM1 and NM2 likely share a common ancestor with NM L93/4286, a serogroup C invasive strain from the United Kingdom (*12*). A rooted tree constructed by the neighbor-joining method indicated that NM1 and NM2 are closely related to FAM18 (Figure) (*13*). Gene-to-gene comparison with FAM18 loci revealed that 29 out of 1,975 loci were missing in >1 of the urethral isolates, although 24 of these were near the end of a contig in either NM1 or NM2. Of the remaining loci, 1,075 (56%) were identical in all three strains, and 1,848 (96%) were identical in NM1 and NM2.

**Figure:**
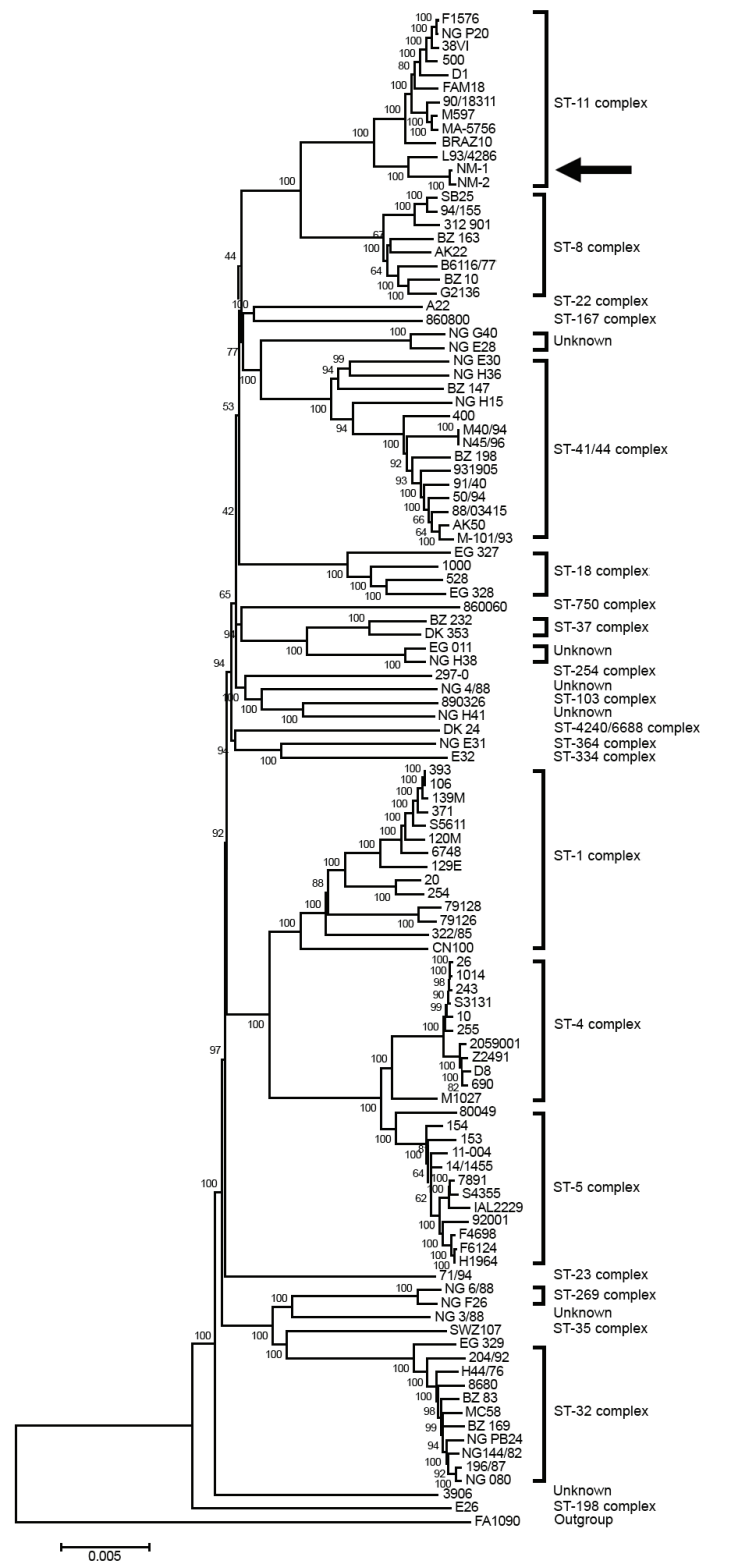
Rooted phylogenetic tree of *Neisseria meningitidis* sequence type 11 urethral isolates from men in Indianapolis, Indiana, USA, 2015–2016, compared with representative serogroup strains of *N. meningitidis*. Tree was inferred by using the neighbor-joining method constructed with MEGA7 (*13*). The percentage of replicate trees in which the associated taxa clustered together in 500 bootstrap tests is indicated next to the branches. The tree is drawn to scale, and branch lengths correspond to evolutionary distances used to infer the phylogenetic tree. Arrow indicates urethritis strains NM1 and NM2. GC strain FA1090 was used as the outgroup. Scale bar indicates nucleotide substitutions per site.

The capsule locus encompasses ≈24 kb in most NM isolates and contains genes that mediate capsule synthesis, transport, assembly and translocation, as well as LPS synthesis. NM1 and NM2 lack *cssC, cssB,* and *cssA* (former designation *siaC, siaB, and siaC* [*14*]), whereas these are intact in NM FAM18 and MC58 (Technical Appendix). Our analysis indicated that these genes are highly conserved in diverse isolates from *N. meningitidis* serogroups B, C, W, and Y, but are also absent in gonococcal strain FA1090.

To estimate the prevalence of presumptive NM urethritis at BFC, we retrospectively examined 107 cases from men seen between 2013 and 2016 who had Gram stain results positive for WBCs and GNID but who had negative GC NAATs. Rates of negative GC NAATs among men with positive Gram stains were 12/436 (2.8%) in 2013, 8/552 (1.4%) in 2014, 37/533 (6.9%) in 2015, and 50/510 (9.8%) in the first 3 quarters of 2016, indicating a significant increase in these cases during the study interval by both a χ^2^ test (p<0.0001) comparing the 4 years and the Mantel-Haenszel test for a trend over time (p = 0.005). Analysis of cases before 2013 was not possible because of the lack of discrimination in the NAAT used at the clinic.

Similar to the case patients recently reported from Ohio and Michigan (*11*), our 37 case-patients in 2015 had an average age of 32 (range 20–61) years and were symptomatic with either discharge (n = 35) or dysuria (n = 2) and predominantly heterosexual (n = 34) (Table). Most received oral sex (n = 35) and were HIV negative (n = 32/32 tested). 

**Table:** Characteristics of 87 men with presumed urethral *Neisseria meningitidis* infection seen at the Bell Flower Clinic, Indianapolis, Indiana, USA, January 1, 2015–September 30, 2016*

Characteristic	**No. (%) persons**	
	2015	2016
Race		
White	6 (16)	5 (10)
Black	29 (78)	45 (90)
Other	2 (5)	0
Ethnicity		
Non-Hispanic	35 (95)	50 (100)
Hispanic	2 (5)	0
Self-identified sexual orientation		
Heterosexual	34 (92)	48 (96)
Homosexual	2 (5)	1(2)
Bisexual	1 (3)	0
Modes of sexual contact†		
Insertive vaginal intercourse	33 (89)	45 (90)
Insertive oral sex	35 (95)	44 (88)
Receptive oral sex	19 (51)	33 (66)
Insertive anal intercourse	1 (3)	1 (2)
Receptive anal intercourse	1 (3)	1 (2)
Symptoms		
Discharge	35 (95)	49 (98)
Dysuria	2 (5)	0
Discharge/dysuria	37 (100)	49 (98)
No discharge/dysuria	0	1 (2)‡
Most recent HIV status		
Negative, documented or self-reported	32 (86)	45 (90)
Not tested and unknown	5 (14)	5 (10)
Exchange sex for drugs or money		
Yes	2 (5)	0
No	33 (89)	49 (98)
Unknown	2 (6)	1 (2)
Noninjection recreational drug use, excluding alcohol, preceding 60 d		
Yes	17 (46)	31 (62)
No	18 (49)	19 (38)
Unknown	2 (5)	0
Treatment provided		
Ceftriaxone plus azithromycin	34 (92)	49 (98)
Ceftriaxone plus doxycycline	1 (3)	0
Other	2 (5)	1 (2)
Urethral coinfection with *Chlamydia trachomatis* by NAAT		
Positive	6 (16)	8 (16)
Negative	31 (84)	42 (84)

*NAATs, nucleic acid amplification tests. †One man had none. ‡Had irritated meatus.

## Conclusions

Together, our results and those of Bazan (*11*) suggest that *N. meningitidis* ST11 could be a notable emerging cause of nongonococcal urethritis; more extensive sequencing and comparisons of recent *N. meningitidis* ST11 isolates from around the United States are underway. However, our results support the observation that both the rates and geographic distribution of *N. meningitidis*–associated urethritis cases in the United States are increasing. Sequencing additional isolates should clarify whether these isolates correspond to an emerging urethrotropic clade of *N. meningitidis*. Retrospective comparisons of NAAT-negative, GNID-positive urethritis case rates might also help discern whether these cases have been increasing elsewhere. As fewer clinics perform routine Gram staining, a serious concern is that infections with urethral discharge, coupled with a negative gonococcus-specific NAAT, could be misdiagnosed as a *Chlamydia* or *Trichomonas* infection. We speculate that an increased number of *N. meningitidis* cases may occur if we limit our clinical diagnoses solely on the results of current diagnostic methods, thereby causing asymptomatic cases to go untreated. Therefore, continued investigation into diagnostic methods targeting urethral specific *N. meningitidis* isolates is pressing, to control the transmission of sexually transmitted *N. meningitidis*. Finally, we note that because the meningococcal *ctrA* gene is highly conserved in NM1 and NM2, they should be able to be differentiated from gonococcus by using meningococcal *ctrA* reverse transcription PCR assay(*15*).

Technical Appendix Results of whole-genome sequencing of *Neisseria meningitidis* isolates NM1 and NM2 and figure showing gene neighborhoods for capsule gene clusters of reference strain *N. meningitidis* serogroup C FAM18 and urethral strains NM1 and NM2.

## References

[1] Feldman HA. Meningococcus and gonococcus: never the Twain—well, hardly ever. N Engl J Med. 1971;285:518–20. 10.1056/NEJM197108262850914 4997666

[2] Carpenter CM, Charles R. Isolation of meningococcus from the genitourinary tract of seven patients. Am J Public Health Nations Health. 1942;32:640–3. 10.2105/AJPH.32.6.640 18015632 PMC1526854

[3] Murray E. Meningococcus infections of the male urogenital tract and the liability to confusion with gonococcus infection. Urol Cutaneous Rev. 1939;43:739–41.

[4] Janda WM, Bohnoff M, Morello JA, Lerner SA. Prevalence and site-pathogen studies of Neisseria meningitidis and N gonorrhoeae in homosexual men. JAMA. 1980;244:2060–4. 10.1001/jama.1980.03310180026026 6776296

[5] Nebreda T, Campos A, Merino FJ. Urethritis caused by *Neisseria meningitidis* serogroup C. Clin Microbiol Infect. 1999;5:57–60. 10.1111/j.1469-0691.1999.tb00101.x 11856216

[6] Rodriguez CN, Rodriguez-Morales AJ, Garcia A, Pastran B, Rios A, Calvo A, et al. Quinolone and azithromycin-resistant *Neisseria meningitidis* serogroup C causing urethritis in a heterosexual man. Int J STD AIDS. 2005;16:649–50. 10.1258/0956462054944363 16176640

[7] Gregory JE, Crook R, Keeler G. Urethritis attributable to *Neisseria meningitidis*, group X: a case report. J Natl Med Assoc. 1979;71:845–6. 116007 PMC2537472

[8] Hayakawa K, Itoda I, Shimuta K, Takahashi H, Ohnishi M. Urethritis caused by novel *Neisseria meningitidis* serogroup W in man who has sex with men, Japan. Emerg Infect Dis. 2014;20:1585–7. 10.3201/eid2009.140349 25154021 PMC4178410

[9] Hagman M, Forslin L, Moi H, Danielsson D. Neisseria meningitidis in specimens from urogenital sites. Is increased awareness necessary? Sex Transm Dis. 1991;18:228–32. 10.1097/00007435-199110000-00006 1771476

[10] Shanmugaratnam K, Pattman RS. Acute urethritis due to *Neisseria meningitidis.* Genitourin Med. 1989;65:401–2. 2515149 10.1136/sti.65.6.401-b PMC1194419

[11] Bazan JA, Peterson AS, Kirkcaldy RD, Briere EC, Maierhofer C, Turner AN, et al. Notes from the field: Increase in *Neisseria meningitidis*–associated urethritis among men at two sentinel clinics—Columbus, Ohio, and Oakland County, Michigan, 2015. MMWR Morb Mortal Wkly Rep. 2016;65:550–2. 10.15585/mmwr.mm6521a5 27254649 PMC5390329

[12] Bratcher HB, Corton C, Jolley KA, Parkhill J, Maiden MC. A gene-by-gene population genomics platform: de novo assembly, annotation and genealogical analysis of 108 representative *Neisseria meningitidis* genomes. BMC Genomics. 2014;15:1138. 10.1186/1471-2164-15-1138 25523208 PMC4377854

[13] Kumar S, Stecher G, Tamura K. MEGA7: Molecular Evolutionary Genetics Analysis version 7.0 for bigger datasets. Mol Biol Evol. 2016;33:1870–4. 10.1093/molbev/msw054 27004904 PMC8210823

[14] Harrison OB, Claus H, Jiang Y, Bennett JS, Bratcher HB, Jolley KA, et al. Description and nomenclature of *Neisseria meningitidis* capsule locus. Emerg Infect Dis. 2013;19:566–73. 10.3201/eid1904.111799 23628376 PMC3647402

[15] Corless CE, Guiver M, Borrow R, Edwards-Jones V, Fox AJ, Kaczmarski EB. Simultaneous detection of *Neisseria meningitidis, Haemophilus influenzae*, and *Streptococcus pneumoniae* in suspected cases of meningitis and septicemia using real-time PCR. J Clin Microbiol. 2001;39:1553–8. 10.1128/JCM.39.4.1553-1558.2001 11283086 PMC87969

