# Supplementary material for: Neisseria meningitidis ST11 Complex Isolates Associated with Nongonococcal Urethritis, Indiana, USA, 2015–2016
Source: Emerg Infect Dis. 2017 Feb;23(2):336–9. doi: 10.3201/eid2302.161434 (PMC5324800; doi:10.3201/eid2302.161434)
Supplement: Technical Appendix — Results of whole-genome sequencing of Neisseria meningitidis isolates NM1 and NM2 and figure showing gene neighborhoods for capsule gene clusters of reference strain N. meningitidis serogroup C FAM18 and urethral strains NM1 and NM2. [file 16-1434-Techapp-s1.pdf]

Article DOI: http://dx.doi.org/10.3201/eid2302.161434

Neisseria meningitidis ST11 Complex Isolates Associated with Nongonococcal Urethritis, Indiana, USA, 2015–2016

Technical Appendix

Additional Methods

Genomes of NM1 and NM2 were paired-end 250-bp sequenced on an Illumina MiSeq, generating a range of 0.67 to 0.75 million reads, yielding ≈600X coverage. The genomes were assembled using SPAdes (*1*), resulting in assemblies consisting of 147 to 174 contigs, and were ordered against the genome of *N. meningitidis* strain MC58 using Mauve Contig Mover (*2*), yielding draft genomes of ~2.14 Mb [GenBank accession nos: (NM1) LXLA00000000, (NM2) LXLB00000000], and an average gonococcal content of 51.7%. RAST (*3*) annotated 2,727 features, including protein-encoding genes and RNAs, and annotation using default multilocus sequence typing (MLST) (*fetA*, *fHbp*, *penA*, *porA*, *porB*, *nhba*, *nadA*), ribosomal MLST, and core-genome MLST schemes (*4*), all indicated that the 2 genomes were from closely-related strains in the ST11 complex, with fine type PorA VR 1.5–1, PorA2 10–8; ST11 (CC11) (*5*). Both NM1 and NM2 had the same PorB type as NM serogroup C strain FAM18 (FAM 18) (*6*), but different FetA F3–6, *fHbp*, and *nhba* types (*7–9*). These genomes also contained the *penA* allele 316, *rpoB* allele 8, and *gyrA* allele 4. ResFinder (*10*) identified no additional acquired antimicrobial resistance genes which was consistent with antimicrobial susceptibility testing results (data not shown).

The capsule polysaccharide structures of meningococci dictate the serogroup identity of individual *N. meningitidis* strains. We compared the FAM18, NM1, and NM2 capsule gene clusters, and found that both NM1 and NM2 were missing three genes, *cssC, cssB,* and *cssA,* (formerly *siaC, siaB, siaA*), which are responsible for cytidine-5′-monophosphate-N-acetylneuraminic acid synthesis, a sialic acid derivative. However, the *ctrG* gene of both NM1 and NM2, which encodes an essential protein involved in enabling the correct expression of

sialic acid polysaccharides, and surface translocation of sialic capsules, was intact (Technical Appendix Figure).

References

1. Bankevich A, Nurk S, Antipov D, Gurevich AA, Dvorkin M, Kulikov AS, et al. SPAdes: a new genome assembly algorithm and its applications to single-cell sequencing. J Comput Biol. 2012;19:455–77. PubMed http://dx.doi.org/10.1089/cmb.2012.0021

2. Rissman AI, Mau B, Biehl BS, Darling AE, Glasner JD, Perna NT. Reordering contigs of draft genomes using the Mauve aligner. Bioinformatics. 2009;25:2071–3. PubMed http://dx.doi.org/10.1093/bioinformatics/btp356

3. Aziz RK, Bartels D, Best AA, DeJongh M, Disz T, Edwards RA, et al. The RAST Server: rapid annotations using subsystems technology. BMC Genomics. 2008;9:75. PubMed http://dx.doi.org/10.1186/1471-2164-9-75

4. Jolley KA, Maiden MCJ. BIGSdb: Scalable analysis of bacterial genome variation at the population level. BMC Bioinformatics. 2010;11:595. PubMed http://dx.doi.org/10.1186/1471-2105-11-595

5. Russell JE, Jolley KA, Feavers IM, Maiden MC, Suker J. PorA variable regions of *Neisseria meningitidis*. Emerg Infect Dis. 2004;10:674–8. PubMed http://dx.doi.org/10.3201/eid1004.030247

6. Tanabe M, Nimigean CM, Iverson TM. Structural basis for solute transport, nucleotide regulation, and immunological recognition of *Neisseria meningitidis* PorB. Proc Natl Acad Sci U S A. 2010;107:6811–6. PubMed http://dx.doi.org/10.1073/pnas.0912115107

7. Thompson EA, Feavers IM, Maiden MC. Antigenic diversity of meningococcal enterobactin receptor FetA, a vaccine component. Microbiology. 2003;149:1849–58. PubMed http://dx.doi.org/10.1099/mic.0.26131-0

8. Seib KL, Scarselli M, Comanducci M, Toneatto D, Masignani V. *Neisseria meningitidis* factor H-binding protein fHbp: a key virulence factor and vaccine antigen. Expert Rev Vaccines. 2015;14:841–59. PubMed http://dx.doi.org/10.1586/14760584.2015.1016915

9. Serruto D, Spadafina T, Ciucchi L, Lewis LA, Ram S, Tontini M, et al. *Neisseria meningitidis* GNA2132, a heparin-binding protein that induces protective immunity in humans. Proc Natl Acad Sci U S A. 2010;107:3770–5. PubMed http://dx.doi.org/10.1073/pnas.0915162107

10. Zankari E, Hasman H, Cosentino S, Vestergaard M, Rasmussen S, Lund O, et al. Identification of acquired antimicrobial resistance genes. J Antimicrob Chemother. 2012;67:2640–4. PubMed http://dx.doi.org/10.1093/jac/dks261

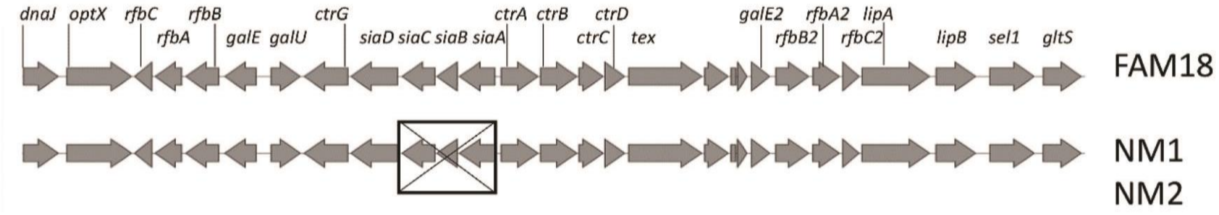

Technical Appendix Figure. Gene neighborhoods for capsule gene clusters of reference strain *Neisseria meningitidis* serogroup C FAM18 and urethral strains NM1 and NM2. Gene synteny is observed within much of the capsule gene cluster, except that the *cssA-C* genes are missing in NM1 and NM2.
